# Integrated mRNA and microRNA transcriptome analyses reveal regulation of thermal acclimation in *Gymnocypris przewalskii*: A case study in Tibetan Schizothoracine fish

**DOI:** 10.1371/journal.pone.0186433

**Published:** 2017-10-18

**Authors:** Cunfang Zhang, Chao Tong, Fei Tian, Kai Zhao

**Affiliations:** 1 Key Laboratory of Adaptation and Evolution of Plateau Biota, Laboratory of Plateau Fish Evolutionary and Functional Genomics, Qinghai Key Laboratory of Animal Ecological Genomics, Northwest Institute of Plateau Biology, Chinese Academy of Sciences, Xining, China; 2 University of Chinese Academy of Sciences, Beijing, China; Chinese Academy of Sciences, CHINA

## Abstract

Environmental acclimation is important episode in wildlife occupation of the high-altitude Tibetan Plateau (TP). Transcriptome-wide studies on thermal acclimation mechanism in fish species are rarely revealed in Tibetan Plateau fish at high altitude. Thus, we used mRNA and miRNA transcriptome sequencing to investigate regulation of thermal acclimation in larval Tibetan naked carp, *Gymnocypris przewalskii*. We first remodeled the regulation network of mRNA and miRNA in thermal acclimation, and then identified differential expression of miRNAs and target mRNAs enriched in metabolic and digestive pathways. Interestingly, we identified two candidate genes contributed to normal skeletal development. The altered expression of these gene groups could potentially be associated with the developmental issues of deformity and induced larval death. Our results have three important implications: first, these findings provide strong evidences to support our hypothesis that *G*. *przewalskii* possess ability to build heat-tolerance against the controversial issue. Second, this study shows that transcriptional and post-transcriptional regulations are extensively involved in thermal acclimation. Third, the integrated mRNA and microRNA transcriptome analyses provide a large number of valuable genetic resources for future studies on environmental stress response in *G*. *przewalskii* and as a case study in Tibetan Schizothoracine fish.

## Introduction

Fish survival is affected by abiotic stresses such as extreme temperatures, salinity, hypoxia and chemical toxicity [1,2], but water temperature is the chief environmental determinant of development, growth, reproduction, behavior, metabolism and geographical distribution [2–4]. As ectotherms, most fish species encounter large temperature variations between day and night and among different seasons, which leads to deleterious consequences when the environmental temperature exceeds species-specific thermal tolerances [5]. Heat stress could influence fish activity and reduce aquaculture production [2], especially as a consequence of global warming [6] which modifies survival, fertility and large-scale migration [7,8]. Animals respond to such environmental changes variously [6,9–12], so it is of both biological and ecological interests to understand the mechanisms underlying the response of fish to heat stress.

Schizothoracine fishes are dominant fish species of the Tibetan Plateau (TP), which has more than 60 species distributed throughout TP altitudes [13,14]. TP aquatic environments are big challenges for aquatic animals and inhospitable due to long-term low temperature, hypoxia, and high salinity [14–16]. Unlike other Schizothoracine fishes, Tibetan naked carp *Gymnocypris przewalskii* is one of the best-studied Schizothoracine species, making it a model aquatic animal organism for studying speciation and environmental adaptation [16–20]. Lake Qinghai is the largest salt lake of China. It is formed during the creation of the TP, which produces the hostile aquatic environment [13]. Consequently, many ancestral endemic fish species were extinct with the exclusive exception of the *G*. *przewalskii*, [13,21] which has a remarkable adaptive capacity that contributed to its adaptation to the Lake Qinghai [16,22]. In addition, previous data show that *G*. *przewalskii* could not tolerate the high water temperature exceeded 30°C, which triggered high mortality in field condition [14]. While, another evidence showed that this cold-water fish species could survive under severe heat exposure (30°C) and the optimum farming temperature of 24°C contributed to a positive impact on the growth rate in the aquaculture industry [19]. Nevertheless, this controversial issue on the thermal acclimation of *G*. *przewalskii* has not been experimentally investigated, and the regulation of mechanism responsible for heat stress in Tibetan Schizothoracine fishes also has been rarely revealed.

Many studies have focused on thermal acclimation in animals [20–25], and suggest that larval and later developmental stages are more sensitive to temperature change than adults [26,27], which allows study of fish responses to heat stress. RNA sequencing technology has been successfully used to reveal the heat (cold) stress response mechanism in larvae of several fish species [5,28,29]. The microRNAs (miRNAs) are a family of endogenous RNAs that regulated gene expression in a sequence-specific manner. The miRNAs are usually 22 nucleotides long with a seed region (2–8 bp) that binds to the 3’ untranslated region (UTR) of a targeted mRNA, signaling it for the degradation. The miRNAs have diverse expression patterns and regulate developmental and physiological processes [30]. Previous studies have confirmed the mechanisms of miRNAs in animal responses to both biotic and abiotic stresses [31–33], while our understanding of the molecular mechanism of thermal acclimation regulated by both mRNA and miRNA in fish remains limited, especially in highland fish species. The potential thermal acclimation of the cold-water fish *G*. *przewalskii* has always been controversial [14,23]. Here we hypothesize that *G*. *przewalskii* could survive and resist heat stress against the controversial issue. Thus, we used the *G*. *przewalskii* larvae as experiment materials, and assessed mRNA and miRNA transcriptomes to investigate the regulation of thermal acclimation in *G*. *przewalskii*.

## Materials and methods

### Animals heat treatment and statistical analysis

**Fig 1:**
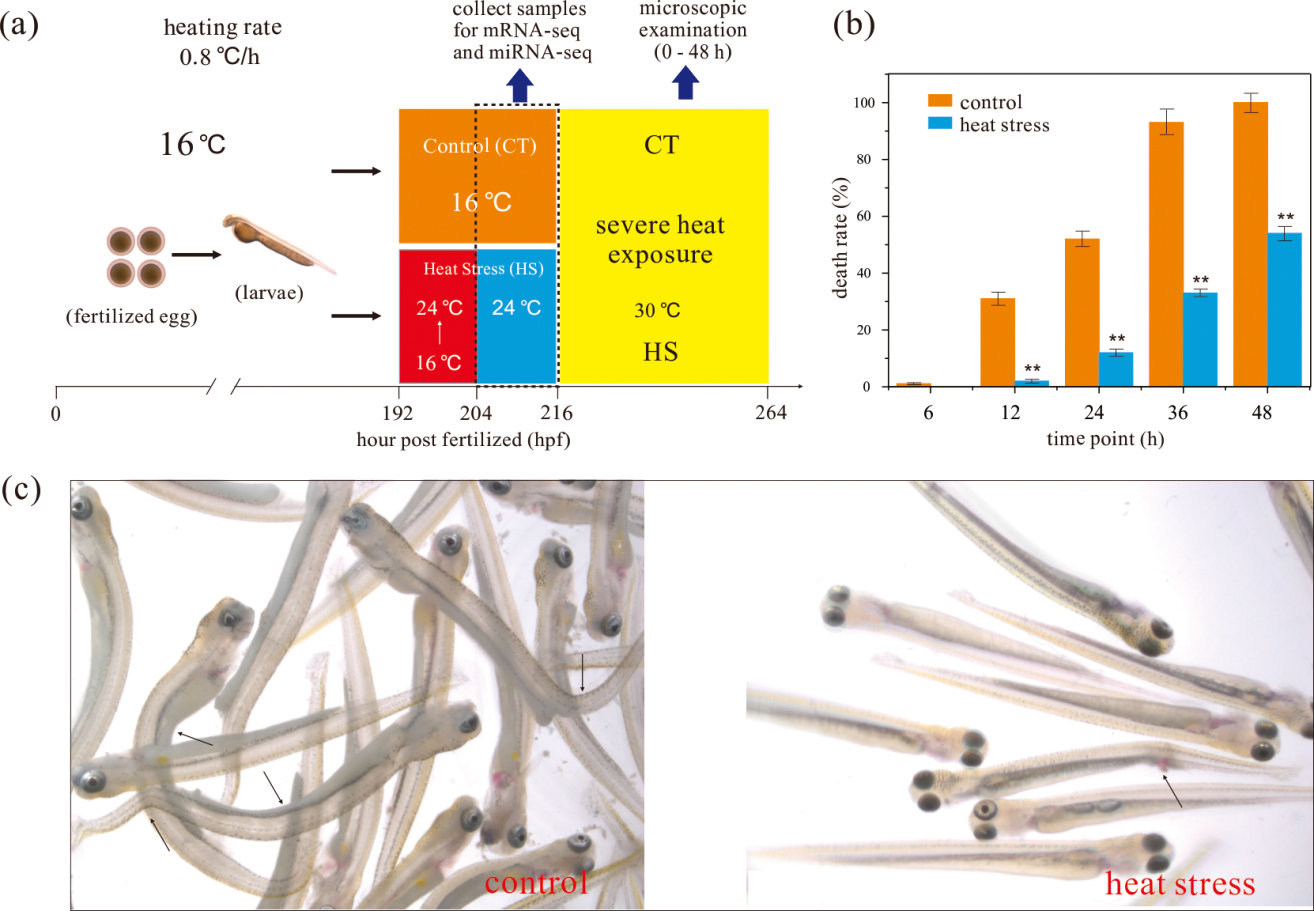
Establishment of heat stress resistance in *G*. *przewalskii* after exposure to mid-high temperature under further severe heat stress. (a) Schematic diagram of heat exposure experiment. *G*. *przewalskii* embryos were incubated at 16°C from fertilization to 192 hpf (hatching out). Larvae at 192 hpf were exposed to heat stress at 24°C (HS group) and the controls (CT group) were maintained at 16°C. Samples for RNA-seq and miRNA-seq were collected at 216 hpf. After a comparative treatment for 12 h, larvae from both groups were further exposed to severe heat stress (30°C) for 48 h. The heating rate is ~ 0.8°C per hour. Images of larvae were taken by using stereomicroscope from Zeiss SteREO DiscoveryV12 with a color CCD camera. Death rates of larvae from both groups were recorded respectively. (b) Bar plot indicates the death rates of larvae from HS and CT groups at different time. HS and CT groups were colored by orange and blue, respectively. Data was shown as mean ± standard error of the mean (n = 3). “**” above error bars indicate *p* < 0.01. (c) Samples from HS and CT groups were exposed to 30°C for 48 h. Image of larvae were also taken by using Zeiss SteREO DiscoveryV12. Red arrowheads indicate representative malformed and dead larvae.

All animal experiments were approved by the Animal Care and Use Committees of the Northwest Institute of Plateau Biology, Chinese Academy of Sciences and the Agriculture Department of Qinghai Province, China. The present study is based on wild-caught *G*. *przewalskii* that were collected in the Lake Qinghai in May 2014 and provided by the National Rescue Center of Qinghai Lake Naked Carp (Xining, Qinghai, China). All the sample were subsequently reared under laboratory conditions. The artificial insemination is based on a wild-caught female and a male of *G*. *przewalskii*. Fertilized eggs were collected and transferred to another re-circulating aerated freshwater system (12 h day/12 h night, 16°C). At 192 hour post fertilization (hpf), all the larvae of *G*. *przewalskii* were hatched out (n = 600, Fig 1A). This developmental stage allows a reseasonable period of post-hatching and no need for feeding, which provided suitable materials for environmental stress experiment [5,29]. Then fish larvae at 192 hpf were separated into two groups (300 larvae per group) and immediately transferred into six dishes (100 larvae per dish). Control group (CT) were continued to maintain at 16°C. Heat stress groups (HS) were exposed to 24°C of 12 hours with heating rate of ~ 0.8°C/h (Fig 1A). No embryonic mortality was observed before the treatment. After heat exposure (216 hpf, Fig 1A), fish were anaesthetized with MS-222 (2%, dipping treatment, Sigma, St. Louis, MO). To generated three biological replicates, equal number of fish larvae from CT and HS groups were collected thrice. All the larvae samples were immediately frozen in liquid nitrogen prior to total RNA extraction for RNA library preparation followed by subsequent mRNA and small RNA sequencing.

Then, larval fish from both HS and CT groups were exposed to severe high temperature (30°C) of 48 hours with a same heating rate of ~ 0.8°C/h (Fig 1A). The morphological observation of larvae was recorded with a stereomicroscope system (Zeiss SteREO DiscoveryV12, Germany). In addition, the death rate of larvae in both groups was recorded separately at 6, 12, 24, 36, and 48 h. Statistical analysis was performed by SAS v9.1.3 (SAS Institute Inc., USA). Significant differences between samples were analyzed by T test. Differences were accepted as significant when *p* < 0.05. All data were shown as mean ± standard error of the mean (SEM).

### RNA extraction and library construction

Total RNA of each sample (n = 2 per group) was isolated using TRIzol (Invitrogen, Carlsbad, CA) and purified using a TruSeq RNA Sample Prep Kit V2 (Illumina Inc., San Diego, CA) according to the manufacturer's instructions. The quality and quantity of total RNA samples were assessed with an Agilent 2100 bioanalyzer (Agilent Technologies, Palo Alto, CA) and SDS-PAGE. Approximately 35 μg of total RNA were used for mRNA transcriptome library preparation (4 libraries), and were sequenced with an Illumina HiSeq™ 2500 instrument.

### Bioinformatics analysis of RNA-seq data

RNA-seq raw reads from each library were preprocessed to filter clipped adapter sequences, contaminated sequences, low-quality reads (Q < 20). High-quality reads were assembled to contigs using the *Trinity* program (http://trinityrnaseq.sf.net) with default parameters. Contigs of each assembly were performed with TGICL to produce long and complete unigenes [34], which were generated with a minimum overlap length of 200 bp. The assembled unigene sequences were aligned with a Blast-X search (cut-off E-value of 1e^-6^) in the public NCBI non-redundant (NR), Swiss-Prot protein and eggNOG databases (http://eggnog.embl.de/version_3.0/). Then the unigene was assigned by protein identity with the highest sequence similarity, which was used for functional annotation using the Blast2GO program [35]. Finally pathway assignments were generated using the KEGG database, Kyoto Encyclopedia of Genes and Genomes [36].

### Gene expression analysis

Gene expression values were calculated as reads aligned to gene per kilo base of exon per million mapped reads (RPKM) [37]. Statistical comparison between two libraries was conducted with *DESeq* package in R software [38]. FDR (false discovery rate) < 0.05 was used as the threshold of *p*-value in multiple tests to measure significant gene expression difference [39]. Genes were only considered differently expressed in a given library when the *p* < 0.05 and a greater than two-fold change (absolute value of log_2_ ratio > 1) in expression across libraries was observed.

### Small RNA sequencing and differential expression analysis

Total RNA from larvae (n = 5 per group) was extracted with miRNeasy Mini kit (Qiagen, Germany), and then used for small RNA library construction (4libraries), finally sequenced on an Illumina HiSeq™ 2500 instrument. Notably, the small RNA sequencing was independent of the mRNA transcriptome sequencing and not shared a single lane on the Illumina sequencer.

Similar to the process of RNA-seq, raw reads of small RNA-seq were treated by trimming adapters and removing poor-quality reads. Other RNAs (rRNA, tRNA, snRNA and snoRNA) were removed by blasting against Genbank database (http://blast.ncbi.nlm.nih.gov) and Rfam database (http://sanger.ac.uk/software/Rfam). Clean reads ranging from 15 to 30 nt were searched against known precursor/mature miRNAs in miRBase 19.0 (http://www.mirbase.org/).

Differentially expressed miRNA (DEM) between CT and HS groups were evaluated on a log2-ratio plot. MiRNA expression was first normalized based on total sequencing reads from each library. Criteria for differentially expressed miRNAs were as follows: absolute value of log2 (HS/CT) ≥ 1.0 and having ≥ 1,000 normalized reads in at least one sample.

### miRNA target prediction and regulatory network construction

*In silico* analysis of DEM targets were predicted in DEG using miRanda 4.0 algorithm (microrna.sanger.ac.uk/targets/v4) and *TargetScan* (www.targetscan.org), based on the complementary region between miRNAs and mRNAs and the thermodynamic stability of the miRNA-mRNA duplex. All the miRNA target mRNAs were calculated and clustered by GO terms and KEGG pathway annotations, respectively. Regulatory network predictions for mRNA and miRNA were performed using Cytoscape 3.3.0 (http://www.cytoscape.org/).

### Functional characterization of rapid heat stress response features

Real-time quantitative PCR (RT-qPCR) was used to evaluate the expression of selected DEGs and DEMs. Specific primers were designed with Beacon Designer 8.0 software (S1 Table). Reversed cDNA and miRNA were synthesized with PrimeScript^TM^ RT Kit (Takara, Dalian, China) and miRcute miRNA Isolation Kit (Tiangen, Beijing, China) following the manufacturer's protocols, respectively. RT-qPCR experiments were performed with Power SYBR Green PCR Master Mix Kit (Applied Biosystems, CA, USA) for mRNA and miRcute Plus miRNA qPCR Detection Kit (Tiangen, Beijing, China) for miRNA on ABI ViiA™7 (ABI, CA, USA) instrument. To normalize the expression values, three genes (*β-actin*, *GAPDH* and *EF-1a*) and three miRNA (miR-22a, miR-152 and miR-25) were selected and used as housekeeping control for mRNA and miRNA expression assessments (S1 Table) [40,41]. All samples were analyzed in triplicate as technical replicates and fold changes were calculated using comparative CT method (also known as the 2^−ΔΔCt^ method). Statistical analysis was performed by SAS v9.1.3 (SAS Institute Inc., USA). Significant differences between samples were analyzed by T test. Differences were accepted as significant when *p* < 0.05. All data are shown as mean ± standard error of the mean (SEM).

## Results

### Effect of rapid heat stress on Tibetan naked carp larval development

After larvae exposure to severe high temperature at 30°C (Fig 1A), the death rates of HS and CT groups had diverged. Statistical analysis showed that the death rates of HS are significantly lower than those of CT at 12, 24, 36 and 48 h (*p* < 0.01 in all time points, Fig 1B). The death rates following severe heat exposure for 36 and 48 h reached 93.2% and 100% in the control, while were 33.1% and 54.4% in the HS group. In addition, microscopic observation found that most of larvae in CT group were dead and displayed malformation after exposure to 30°C for 36 h, but the survival larvae in HS group were normal in morphology (Fig 1C). Thus, these finding suggests that Tibetan naked carp larvae develop a preliminary resistance against severe heat stress (30°C) after exposure to 24°C.

### Differential expression of mRNAs and miRNAs

**Fig 2:**
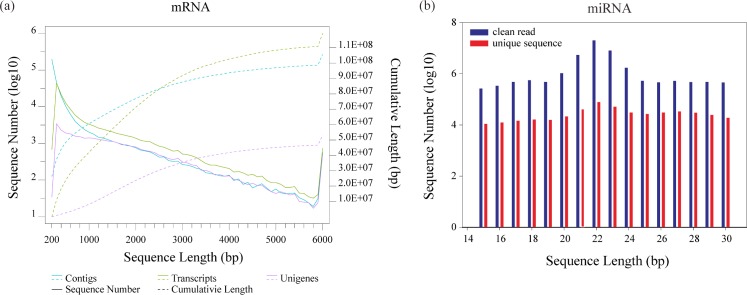
Length distribution of contigs, transcripts and unigenes of mRNA and miRNA in Tibetan naked carp. (a) Distribution of assemblies (contigs, transcripts and unigenes). The left y-axis and solid lines are the distributions of number (log10-transformed) of assemblies in each 100-bp bin, while the right y-axis and dashed lines are the cumulative curves for each assembly. (b) Distributions of sequence number of clean and unique sequence within miRNA-seq. All the plots were carried out with ggplot2 in R version 3.2.1.

**Fig 3:**
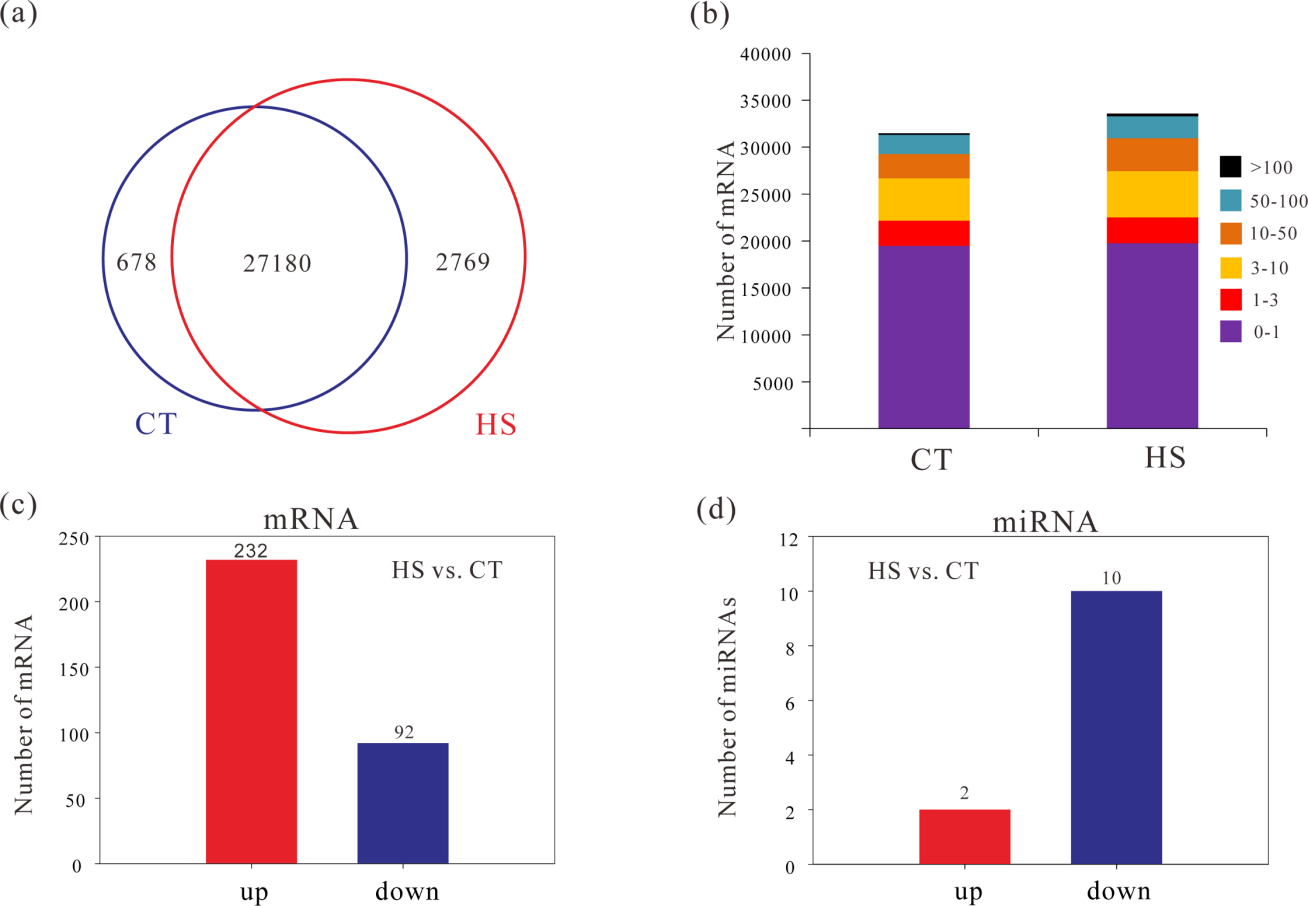
Comparisons between biological replication data sets of heat stress group (HS) and control groups (CT). (a) Number of expressed genes within HS and CT; (b) RPKM range of specific expressed genes between HS and CT; (c) Significantly up- and down-regulated mRNAs between HS and CT comparisons; (d) Significantly up- and down regulated miRNAs between HS and CT comparisons.

From six larvae RNA-seq datasets (three biological replicates each group) of Tibetan naked carp larvae at 16°C (CT) and 24°C (HS) were obtained after Illumina sequencing. From 92 million raw reads, 77 million clean reads were identified (Fig 2A and S2 Table). High-quality reads were assembled into 30,672 unigenes by *Trinity* [42], annotated and mapped to public databases (S3 Table). The high Pearson correlation coefficient suggested good repeatability within each group (S1 Fig). Then the unigene expression (RPKM) data were calculated between CT and HS with *DESeq* [43], which showed that 27,180genes were expressed in both CT and HS as well as 678 genes were specifically expressed in CT (Fig 3A and S3 Table). With heat stress, 2,769 genes are expressed (Fig 3A and S3 Table). The comparison of cumulative RPKM values of all genes in CT and HS groups indicates that HS gene expression is greater than in CT (Fig 3B). Finally, 324 genes with an absolute log2-ratio value ≥1 were up- (232) or downregulation (92) when HS and CT were compared, which were identified as DEG (Fig 3C, S2 Fig and S4 Table).

miRNA sequencing generated more than 60 million raw reads to yield 47 million clean reads which were narrowed to 41,585,187 reads ranging from 15 to 30 nt (Fig 2B and S4 Table). The Pearson correlation analysis also confirmed that the good repeatability among samples within group (S1 Fig). Data from CT and HS replicates were represented by more than 10 million clean reads, which was sufficient for the subsequent quantitative analysis of miRNAs. CT and HS shared 1,114 expressed miRNAs, but only 2 miRNAs were upregulated and 10 miRNAs were downregulated when comparing HS and CT groups (Fig 3D, S2 Fig and S5 Table) and these were identified as DEMs.

### DEGs associated with normal skeletal development

**Fig 4:**
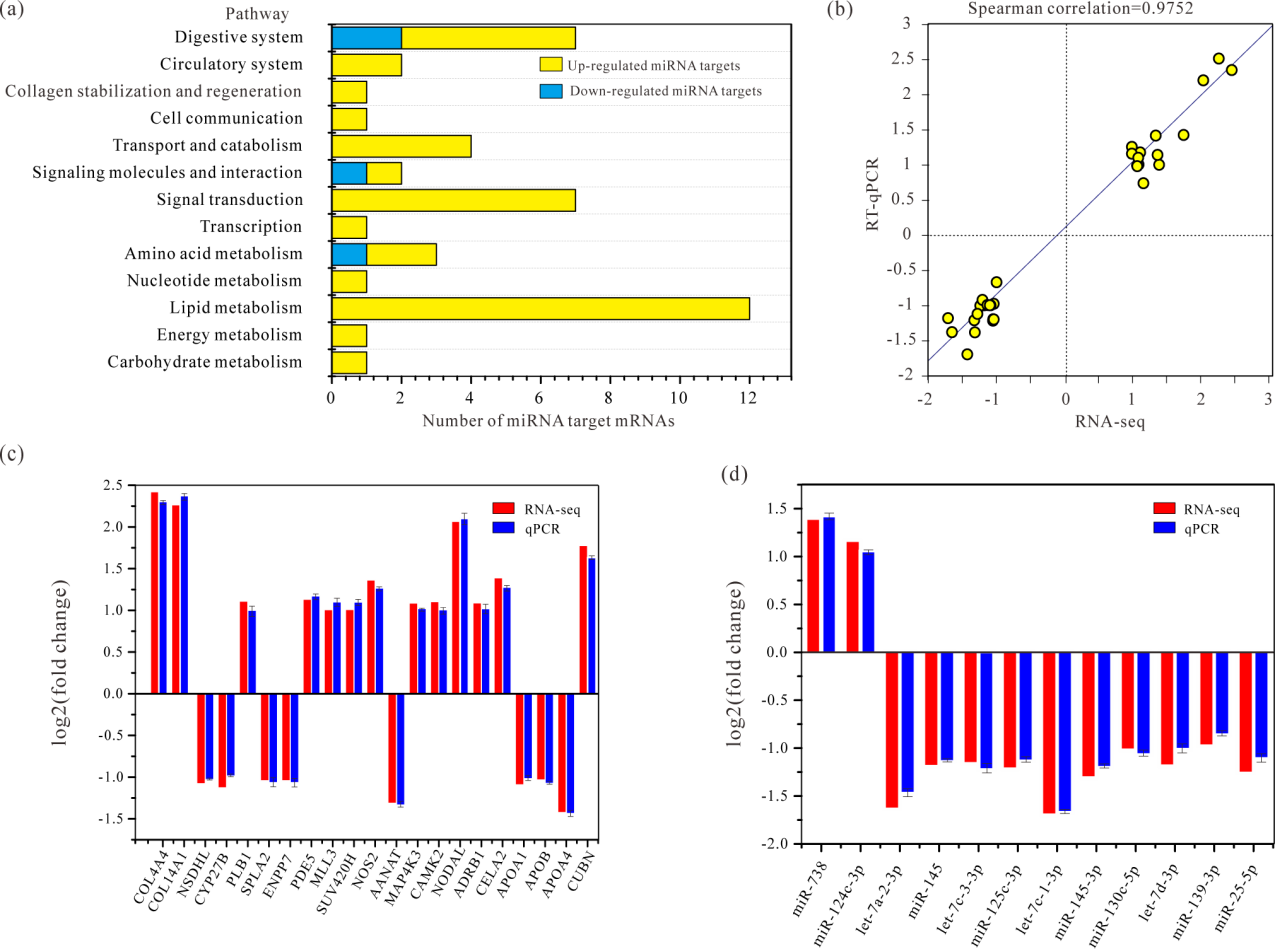
Statistics comparisons of KEGG pathway and validation of RNA-seq data by RT-qPCR. (a) Comparisons of changes in miRNAs targets involved in the KEGG pathway. (b) Fold changes of gene expression detected by RNA-seq were plotted against the data of RT-qPCR. The reference line indicates the linear relationship between the results of RNA-seq and RT-qPCR. The correlation between RNA-seq and RT-qPCR data was analyzed by Spearman’s rho test (r^2^ = 0.9752). (c) Expression comparisons of selected DEMs target genes according to RNA-seq and RT-qPCR. Bars of RNA-seq and RT-qPCR date were colored by red and purple, respectively. Data of RT-qPCR were shown as mean ± standard error of the mean (n = 3). (d) Comparisons of significant changes in miRNAs between HS and CT groups by RNA-seq and qPCR.

Collagen stabilization and regeneration could contribute to maintain the normal skeletal development in the early development of larval fish [44,45]. Morphological evidences indicated that severe heat exposure (30°C) had induced malformation of fish larvae as the abnormal skeletal development, and also triggered high death rate in CT group (Fig 1B and 1C). Intriguingly, we found that two DEGs *collagen alpha-4 (IV) chain* (COL4A4) and *collagen alpha-1(XIV) chain* (COL14A1) that were involved into collagen stabilization and regeneration were significantly up-regulated in response to heat stress (Table 1 and Fig 4A). The malformation observed under heat stress was presumably associated with the altered expression of *COL4A4* and *COL14A1*, whose function was to maintain the normal skeletal development.

**Table 1:** DEGs of pathway within Tibetan naked carp exposure to rapid heat stress.

Protein	Sub-catalog	Log_2_(HS/CT)	Description
*Collagen stabilization and regeneration*			
COL4A4	collagen catabolic process; extracellular matrix organization	2.487	collagen alpha-4 (IV) chain
COL14A1	collagen binding; extracellular matrix structural constituent	2.296	collagen alpha-1(XIV) chain
*Lipid metabolism*			
NSDHL	Steroid biosynthesis	-1.079*	sterol-4alpha-carboxylate 3-dehydrogenase
CYP27B	Steroid biosynthesis	-1.128*	25-hydroxyvitamin D3 1alpha-hydroxylase
PLB1	Glycerophospholipid metabolis; Arachidonic acid metabolism	1.111	phospholipase B1
SPLA2	Glycerophospholipid metabolis	-1.043*	secretory phospholipase A2
ENPP7	Sphingolipid metabolism	-1.042*	ectonucleotide pyrophosphatase/phosphodiesterase family member 7
*Nucleotide metabolism; Amino acid metabolism*			
PDE5	Purine metabolism	1.133	cGMP-specific 3',5'-cyclic phosphodiesterase
MLL3	Lysine degradation	1.009	histone-lysine N-methyltransferase MLL3
SUV420H	Lysine degradation	1.011	histone-lysine N-methyltransferase SUV420H
NOS2	Arginine and proline metabolis	1.364	nitric-oxide synthase 2
AANAT	Tryptophan metabolism	-1.313*	arylalkylamine N-acetyltransferase
*Signal transduction*			
MAP4K3	MAPK signaling pathway	1.088	mitogen-activated protein kinase kinase kinase kinase 3
CAMK2	Wnt signaling pathway	1.104	calcium/calmodulin-dependent protein kinase
NODAL	TGF-beta signaling pathway	2.067	nodal
*Digestive system*			
ADRB1	Salivary secretion	1.089*	adrenergic receptor beta-1
CELA2	Pancreatic secretion	1.391*	pancreatic elastase 2
APOA1	Fat digestion and absorption	-1.092*	apolipoprotein A1
APOB	Fat digestion and absorption	-1.034*	apolipoprotein B
APOA4	Fat digestion and absorption	-1.424*	apolipoprotein A4
CUBN	Vitamin digestion and absorption	1.777	cubilin

### Lipid metabolism and digestive system pathway genes involved in rapid heat stress response

Lipid metabolism and digestive system pathways are key to fish physiology and maintain health [46]. DEGs involved in signal transduction, digestive system and lipid metabolism pathways were significantly higher than others (Fig 4A). In the tricarboxylic acid (TCA) cycle, the *acylphosphatase* gene involved in pyruvate metabolism, and *phosphatidylinositol-bisphosphatase* gene involved in inositol phosphate metabolism were upregulated after heat stress, but no other gene was modified (Table 1 and S6 Table), suggesting an overall upregulation of the main pathway. DEGs involved in lipid metabolism, such as *secretory phospholipase A2* and *phospholipase B1* genes engaged in glycerophospholipid metabolism, ether lipid metabolism and arachidonic acid metabolism, *ectonucleotide pyrophosphatase/phosphodiesterase family member 7* in sphingolipid metabolism were significantly upregulated. In addition, DEGs involved in the endocrine system, such as *calcium/calmodulin-dependent protein kinase* (CAMK2) that played an essential role in insulin secretion, GnRH signaling pathway, oxytocin signaling pathway and melanogenesis, were upregulated in HS (Table 1 and S7 Table). In the digestive system, *adrenergic receptor beta-1* (ADRB1) gene that was involved in salivary secretion and the *pancreatic elastase 2* (CELA2) gene that was involved in protein digestion and absorption were upregulated. Meanwhile, *apolipoprotein A1* (APOA1), *B* (APOB) and *A4* (APOA4) genes participated in vitamin digestion and absorption were downregulated (Table 1 and S8 Table).

### Regulation network of miRNA and target mRNA

**Fig 5:**
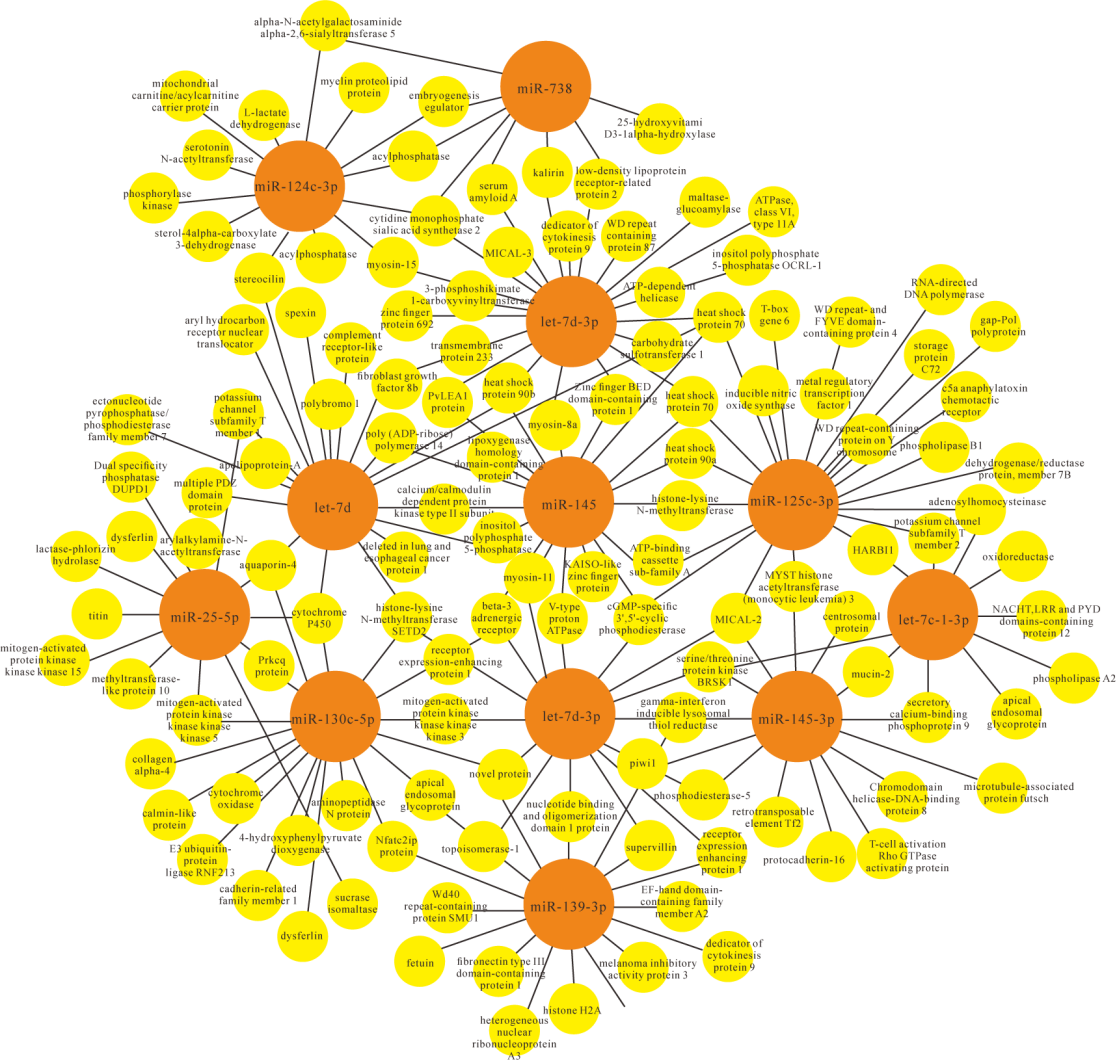
Regulation network of DEMs and targeted DEGs in response to rapid heat stress within Tibetan naked carp. The red circle represents the DEM, and yellow circle represents the DEG, the line represents that there are links between the DEM and target DEG. All the DEM and target DEG were collected and predicted with *Cytoscape* 3.3.0 software. Image was carried out with *Coreldraw* 14.0 software.

Using *in silico* analysis of miRNA target mRNAs with miRanda 4.0 and TargetScan, we obtained a total of 34,258 targeted mRNA and 4,167,259 sites (S9 Table). Mapping the DEM and their targeted mRNA yielded more than 3,000 targeted mRNA. Based on the GO and KEGG annotation (S6 and S8 Tables), many DEM targeted mRNAs were enriched in the digestive system, signal transduction and lipid metabolism pathways, in a good agreement with previous DEG annotation results (S3 Fig). Finally we constructed the network of DEM and their target mRNAs using *Cytoscape* 3.3.0 (Fig 5).

### Validation of RNA-seq data by qPCR

After comprehensive RT-qPCR validation, expression of 21 selected DEGs and 12 DEMs were measured and compared with the expression profiles from RNA-seq analysis. The expression data for these selected DEGs and DEMs detected by RNA-seq and RT-qPCR were displayed in S9 Table and Fig 4. The data from RT-qPCR and RNA-seq exhibited an excellent agreement on both up- and down-regulated genes (Fig 4B). The correlation between RNA-seq and RT-qPCR data was analyzed by Pearson’s correlation coefficient (Fig 4B), and a highly statistical significance (Fig 4C and 4D) was observed.

## Discussion

This study represented the first transcriptome-wide analysis of thermal acclimation in Schizothoracine fish species. Numbers of studies had revealed the thermal acclimation in fish species on a large-scale data level [47–50], while previous studies of thermal (cold) acclimation in Schizothoracine fishes still based on few candidate genes [51,52]. Thanks to the rapid development and decreasing costs of RNA sequencing technologies in recent years, we were able to obtain more than 30,000 genes and 1,000 miRNAs in *G*. *przewalskii*. In addition, the combination of mRNA and miRNA transcriptome sequencing enabled us to obtain a larger number of candidates for thermal acclimation. This study also provided valuable genetic resources for future studies on environmental stress response in Schizothoracine fish.

Most TP lakes are frozen for long periods of time with low water temperature for aquatic organisms [53], and Schizothoracine fish species endemic to highland lakes could survive under this condition [13,14]. *G*. *przewalskii* is a commercially important aquaculture species in Northwestern China, and it inhabits in Lake Qinghai with lengthy period of cold water (September to April) [14,23,53]. Water temperature is the chief environmental determinant of the growth performance of fishes [2–4,46], while it is also the physiological constraint for the ongoing aquaculture industry of this coldwater fish species. In the current study, we focused on the controversial issue of the survival and the growth of *G*. *przewalskii* in high temperature condition [14,23]. Evidences of earlier studies had revealed that thermal experiences of fish at embryonic stages could have dramatic and persistent effects on the postnatal thermal acclimation capacity [42,46], and a number of studies had focused on thermal (cold) acclimation in fish larvae [5,29,48]. In this study, we designed a comparative experiment using *G*. *przewalskii* larvae as materials to investigate the potential thermal acclimation in Tibetan Schizothoracine fishes. Data showed that exposure of *G*. *przewalskii* larvae to 24°C for 12 h increased larval survival under further severe heat stress at 30°C, but the direct exposure to 30°C without a mild high temperature transition caused massive malformation and death. The result was in line with previous studies proposing that *G*. *przewalskii* possessed a rapid heat hardening (RHH) ability to establish a heat tolerance under mild heat stress [5,29]. The current study represented initial evidence of RHH-like responses in *G*. *przewalskii* as well as lays a foundation for studying molecular mechanisms underlying the establishment of RHH in highland larvae in the TP.

How organisms respond to the environment is of increasing importance to biologists and ecologists with the concerning of global climate change [6,54]. Thus, transcriptome studies to address how environmental stimuli affect gene expression can be used to identify different expressed genes (DEG) between HS and CT by mapping unigenes to a pre-assembly reference transcriptome dataset using a *DESeq* package [43]. Specifically, the rapid heat stress triggered and silenced gene expression in *G*. *przewalskii* [29,55]. Interestingly, species-specific expressed genes were chiefly associated with lipid metabolism, indicating that *G*. *przewalskii* might increase their metabolic rate and lipid consumption upon exposure to heat stress. Also, HS and CT were not different with respect to RPKM values, suggesting that *G*. *przewalskii* acclimate to the high temperature and restore homeostasis eventually.

Using a rigorous set of thresholds, we confirmed that 232 genes were up-regulated and 92 genes were downregulated after rapid heat stress exposure. These DEGs are mainly enriched in energy metabolic and digestive system pathways, corresponding well with previous work in the olive flounder [55]. Temperature can affect fish metabolism [2,4,10], and lipids are the chief energy source for most organisms, therefore lipid metabolism is selected to meet energy demands during the larval stage [29,47].

A striking finding of this study is the observation that the rapid heat stress had significant elevated gene expression of *COL4A4* and *COL14A1*, which functions in collagen stabilization and regeneration. Past evidences reveal that the stabilization and regeneration of collage contributes to maintaining normal skeletal development in larval fish early developmental stages [44,45]. Notably, there was significant mortality and deformities in the control fish after a rapid heat shock (16 up to 30°C) by microscopic examination. This observation was in agreement with previous studies during the process of thermal acclimation of other fish species [56–58]. Additionally, *COL4A4* encodes one of the six subunits of type IV collagen, as the major structural component of basement membranes [59], involved into normal skeletal development [44]. Another evidence shows that an increase of *COL14A1* expression contributes to maintain the normal development of mouse axial skeleton [60]. Thus, the heat stress altered expression of these gene groups that could potentially be associated with the developmental issues observed. These findings also indicate a link between heat stresses alter collagen stabilization and regeneration related gene expression and skeletal deformity.

Only two miRNAs in the HS group were upregulated and 10 miRNAs were downregulated. Data show that miRNAs can regulate cell differentiation, organogenesis, development and growth [30,61]. Two *let-7* family members, *let-7d-3p* and *let-7c-1-3p* were downregulated and they are involved in responses to rapid heat stress, which is similar to *let-7c* and *let-7d* expression pattern in response to heat stress in rat small intestine [62]. *Let-7* was subsequently identified as the first known *C elegans* miRNA [63]. *let-7* and its family members are highly conserved across species in sequence and function, such as cell proliferation and growth pathways [64]. In addition, *miR-145* and *miR-125* were uniquely expressed between HS and CT groups and they have significant roles in cell response to stresses such as heat shock and oxidative stress [65]. Furthermore, the function of the remaining eight DEMs are not clearly illustrated, therefore we speculate that these miRNAs are presumably involved in the rapid heat stress response regulation in Tibetan naked carp. Most DEGs described here are involved in metabolic pathways, and DEM target mRNAs are also related to metabolic pathways, suggesting that an important role in the heat stress response.

We predicted and constructed a network diagram of miRNAs and their targets mRNAs by *Cytoscape*. We initially identified more than 100 genes which were potentially targeted by 12 differentially expressed miRNAs according to *in silico* analysis, but most of target mRNAs were not significantly expressed according to HS and CT comparisons. Finally, 56 DEGs targeted by 12 DEMs were identified, including *hsp70*, *hsp90a* and *hsp90b* that were regulated by DEMs in this network, suggesting that miRNAs significantly regulated Tibetan naked carp responses to heat stress [66]. Myosins are a family of ATP-dependent motor proteins with roles in muscle contraction and other motility processes [67], and *myosin 8a*, *-11*and *-15* are in this network, suggesting a link with heat stress response regulation to muscle movement.

## Supporting information

S1 Fig Pearson correlation between samples (n = 2) in each groups (HS and CT). (PDF)

S2 Fig Volcano plot of DEGs and DEMs of Tibetan naked carp response to rapid heat stress. (PDF)

S3 Fig Gene ontology classification of the DEGs and DEMs target genes of Tibetan naked carp response to rapid heat stress. (PDF)

S1 Table Primer list of selected DEGs and DEMs in RT-qPCR verification experiments. (XLS)

S2 Table Overview of sequencing, assembly and analysis of Tibetan naked carp transcriptome. (XLS)

S3 Table Annotation of all Tibetan naked carp unigenes. (XLS)

S4 Table List of statistically significant different expression genes (DEGs). (XLS)

S5 Table The result of conservative miRNA annotation analysis. (XLS)

S6 Table The significantly up/downregulated miRNAs in Tibetan naked carp response to rapid heat stress. (XLS)

S7 Table Enriched KEGG pathways of DEGs in Tibetan naked carp response to rapid heat stress. (XLS)

S8 Table GO terms enriched in up-regulated and down-regulated genes. (XLS)

S9 Table The result of DEMs and targeted DEMs analysis. (XLS)
