# Supplementary figures and images for: Integrated mRNA and microRNA transcriptome analyses reveal regulation of thermal acclimation in Gymnocypris przewalskii: A case study in Tibetan Schizothoracine fish
Source: PLoS One. 2017 Oct 18;12(10):e0186433. doi: 10.1371/journal.pone.0186433 (PMC5646821; doi:10.1371/journal.pone.0186433)

Pearson correlation between samples

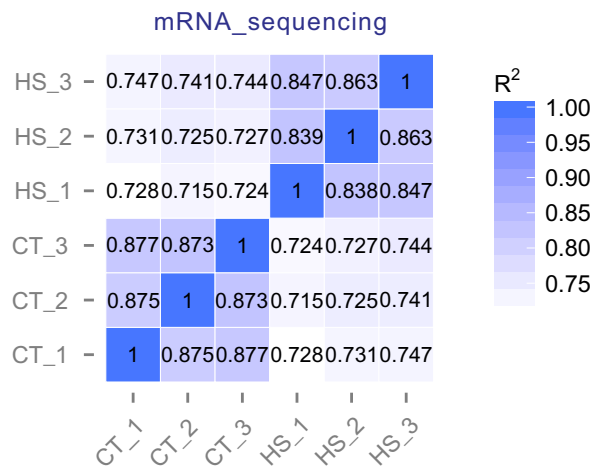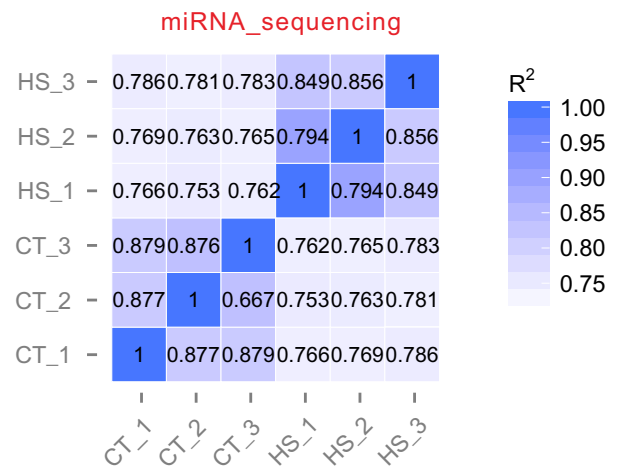

Supplement: S1 Fig — (PDF) [file pone.0186433.s001.pdf]

A

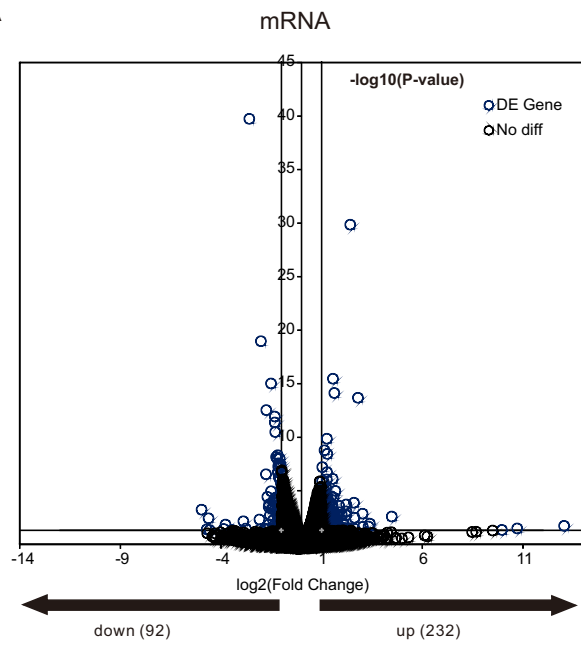

B

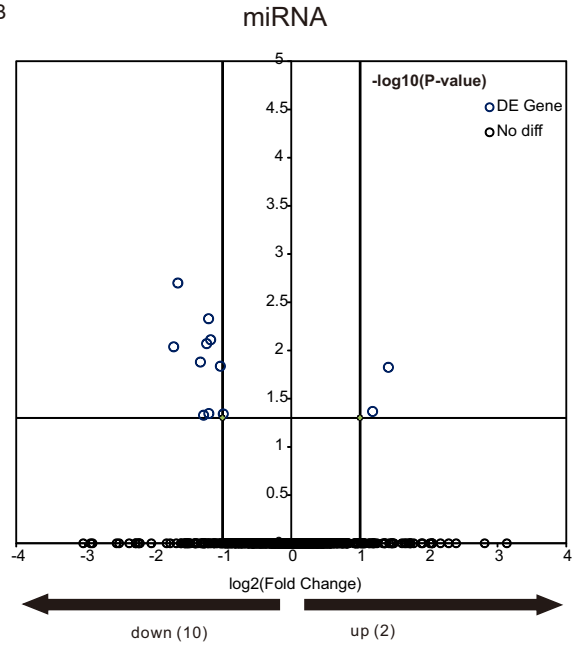

Supplement: S2 Fig — (PDF) [file pone.0186433.s002.pdf]

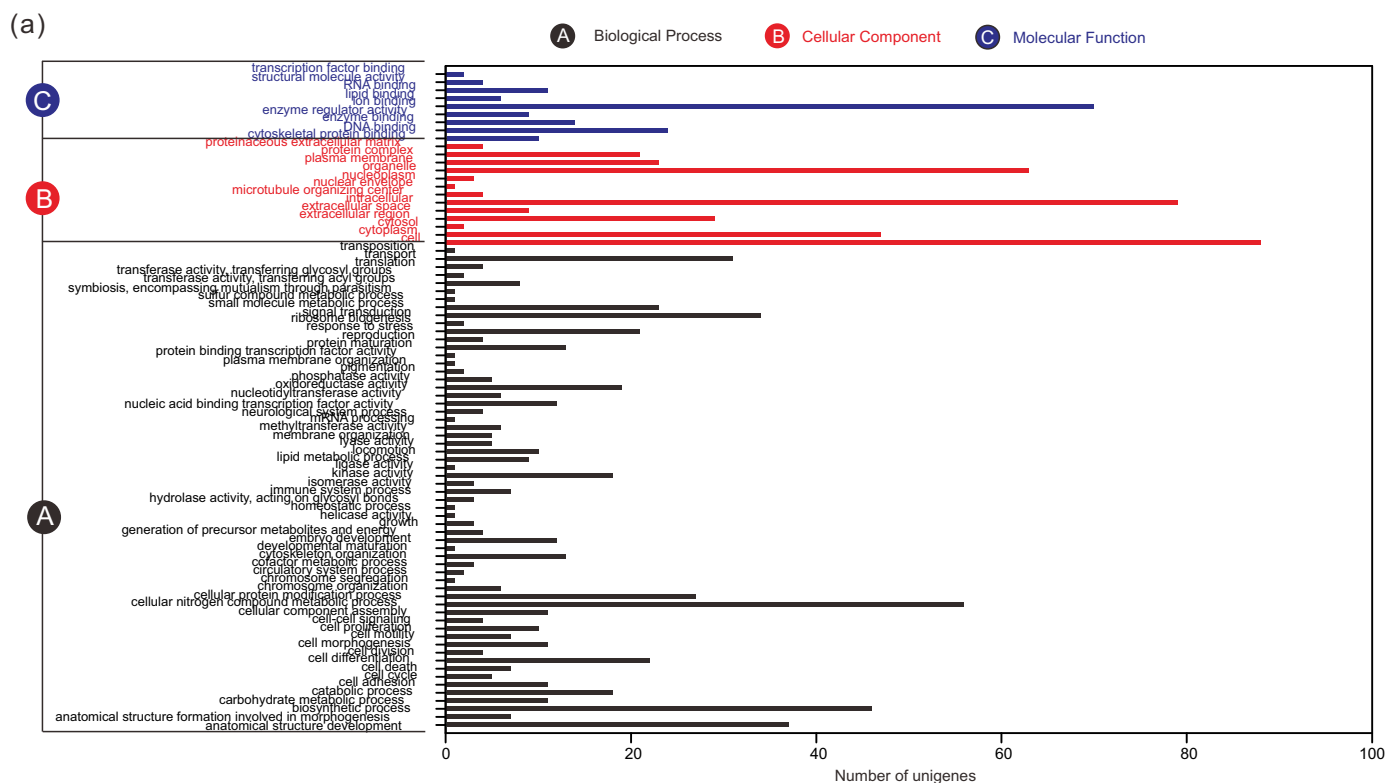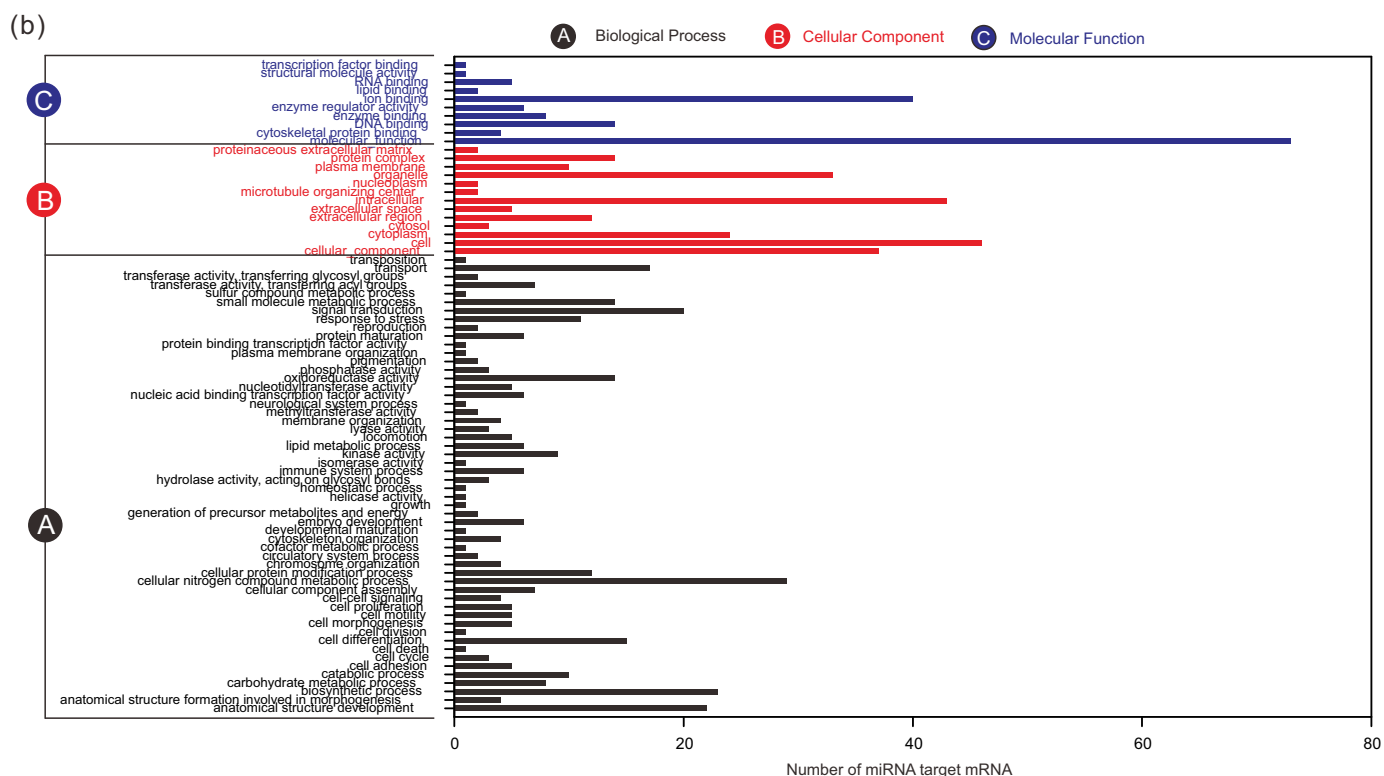

Supplement: S3 Fig — (PDF) [file pone.0186433.s003.pdf]
